# Coping Mechanism among Parents of Children with Autism Spectrum Disorder: A Review

**DOI:** 10.22037/ijcn.v16i2.31518

**Published:** 2022-01-01

**Authors:** Haytham Al-ORAN, Lee KHUAN, Lim Poh YING, Ola HASSOUNEH

**Affiliations:** 1 Department of Child Health Nursing, Faculty of Nursing, Muʼtah University, Jordan.; 2 Department of Nursing & Rehabilitation, Faculty of Medicine and Health Science, Universiti Putra Malaysia, Malaysia.; 3 Department of Community Health, Faculty of Medicine and Health Sciences, Universiti Putra Malaysia, Malaysia.; 4 Department of Child Health Nursing, Princess Muna College of Nursing, Muʼtah University, Jordan.

**Keywords:** Autism spectrum disorder, coping mechanism, coping strategies

## Abstract

This review presented the current literature on coping mechanisms among parents of children with autism spectrum disorder (ASD), focusing on types of coping mechanisms among parents and different coping mechanisms between mothers and fathers of children with ASD.

A search of published literature in English was conducted using Google Scholar, PsycINFO, Medline, Scopus, CINAHL, EBSCO, Springer, Ovid, PubMed, and Cochrane Library up to February 2020.

Overall, 18 articles were relevant to the review. The review included thirteen studies for types of coping mechanisms among parents of children with ASD and five studies for different coping mechanisms between mothers and fathers. Coping mechanisms demonstrated by parents when caring for their child include problem-focused and emotion-focused coping. A comparison between fathers and mothers in our review showed that mothers used emotion-focused coping more than fathers, while fathers used problem-focused coping more frequently than mothers.

The review provides an exciting opportunity to advance our knowledge on types of coping mechanisms and gender difference in using coping mechanisms among parents of children with ASD. The review also sheds new light on developing supportive interventions by healthcare providers to improve coping mechanisms among parents of children with ASD.

## Introduction

Autism spectrum disorder (ASD) is defined as one of the neurological developmental disorders with moderate to severe impairments in social interactions, communication, cognitive development, and repetitive and restricted behaviors (1). Besides, ASD is described as a group of disorders, including autism or autistic disorder, Asperger syndrome, and pervasive developmental disorder. Among these, autism or autistic disorder is the most common type of ASD (1). 

The incidence of ASD varies markedly between countries. The Centers for Disease Control and Prevention estimates that one in 68 children under eight years old is affected by autism. This new estimate is approximately 30% higher than the previous estimate in 2012, with approximately one in 88 children. The incidence rate of ASD in males was five times higher than in females, which was one in 42 boys versus one in 189 girls (2). There is no doubt that the core characteristics of ASD are behavioral problems and socio-communication skill deficits, which lead to significant stress in families with these children (3–5).

To date, numerous studies have revealed that parents of children with ASD experience a higher level of stress than parents of children with other developmental disabilities (6–9). These extensive studies examined factors associated with the high-stress level among parents of children with ASD. Some have found that the parent's gender is strongly associated with the high-stress level (10–14). Further studies have suggested that parenting stress is associated with younger parents and younger male children (15–18). Also, studies have reported that parents experience a higher level of stress in the period right after the diagnostic given (15, 19–20). Studies also reported that parents with lower education levels and monthly income experienced a higher stress level (12,15,21-22). Furthermore, previous studies reported that a high parenting stress level was associated with other factors such as child behavioral problems (23–24) and lack of professional and social support (25–27).

Coping with a stressful situation is challenging (28). According to Lazaras and Folkman (29), coping refers to the behavioral and cognitive abilities to manage and deal with one's internal and external demands in any stressful situation. With this regard, Lazarus and Folkman (29) suggested that a coping mechanism could be either problem-focused or emotion-focused. Problem-focused coping involves identifying the source of the stress and implementing strategies to eliminate or change the stressor. Emotion-focused coping deals more with changing the emotions associated with the stressors. There is evidence that coping plays a crucial role in mastering, reducing, or tolerating stress and determining whether a stressful event is an adaptive or maladaptive process (30–31).

Coping mechanisms that are often demonstrated by parents when caring for their child with ASD include support from family, friends, social support groups, other parents of children with ASD, service providers, advocacy, and religion (32–33). Furthermore, a parent's ability to deal with a high-stress level depends on the effectiveness and quantity of the coping mechanism they employ in managing the demands of stressors associated with a child with ASD (34). This review provided an overview of the evidence for coping mechanisms used by parents of children with ASD.

## Materials & Methods

*Search strategy*

A literature search was performed using the selected databases and keywords and by applying a limit to the search results and the number of articles extracted. To find literature on this topic, electronic medical and healthcare databases , including Google Scholar, PsycINFO, Medline, Scopus, CINAHL, EBSCO, Springer, Ovid, PubMed, and Cochrane Library, were searched. The databases were selected because they covered aspects, such as relevance and key areas, and could provide relevant articles. Many older articles were used due to their value and impact on the study field. This literature review included studies published from 1980 to February 2020. Also, the search was limited to articles presented in the English language. The following keyword search terms were used "Autism spectrum disorder," " Autism," " autistic disorder," "parent mental health," " parenting stress," " coping," " coping mechanism," " coping mechanism," " coping skills," " parents coping style." The advanced search terms were a combination of "Autism spectrum disorder," OR "Autism," OR "autistic disorder," "parent mental health," OR "parenting stress," "coping mechanism," OR "coping strategies," OR "coping skills," OR "parents coping mechanism."

*Inclusion and exclusion criteria*

All the selected articles met the following inclusion criteria: (1) they were published in English; (2) they examined coping mechanisms, coping strategies, or coping skills as their main objectives; (3) they involved quantitative and qualitative approaches. Letters and case reports were excluded.

*Included and excluded studies*

As of February 2020, the search strategy yielded 1041 articles. All the articles were written in the English language with a full text/methodology section following the inclusion and exclusion criteria. However, 55 articles were found to be duplicates. Finally, 18 articles were included in the review. Figure 1 illustrates a flowchart of the process of selecting relevant articles.

## Results

All the identified relevant articles were extensively reviewed to identify themes related to coping mechanisms among parents of children with ASD. Two themes were identified: types of coping mechanisms among the parent of children with ASD and different coping mechanisms between mothers and fathers of children with ASD.

*Types of coping mechanisms among parents of children with ASD*

Research has been conducted on coping mechanisms since the 1980s. Several researchers have attempted to identify types of coping mechanisms among parents of children with ASD. Zablotsky and colleagues (34) reported a strong relationship between emotion-focused coping, such as social support, and parents' psychological health. In an earlier attempt, Sivberg (35) conducted a study to examine the coping level and types of coping mechanisms among 66 parents of children with ASD. The coping mechanisms were assessed using the Ways of Coping Questionnaire (WCQ). The results indicated that parents reported a low level of coping. Additionally, the author found that confrontation, self-control, and escape were the frequently used emotion-focused coping mechanisms by the parents. In the study of Twoy, Connolly, and Novak (36), parents reported that support from families and close friends was frequently used as emotion-focused coping. A survey conducted by Benson (37) showed that mothers of children with ASD used cognitive reframing as emotion-focused coping.

A study was conducted by Smith and colleagues (38) to examine coping mechanisms among 153 parents of children with ASD. The COPE questionnaire, a self-report with a four-point Likert scale, was used to assess the coping mechanisms. The result of the study revealed that mothers of children with ASD reported the highest scores in using active coping, planning, and reframing as problem-focused coping. Also, Kiami and Goodgold (39) conducted a study to identify coping mechanisms and interviewed 70 mothers. In their study, the Coping Health Inventory for Parents (CHIP) questionnaire was used, a self-report form containing 45 items with a score range between 0–4. The authors found that communication with other parents of children with ASD and seeking support from others were the most frequently used problem-focused coping mechanisms among mothers. Another recent study by Pepperell, Paynter, and Gilmor (40) suggested that planning was the most frequently used problem-focused coping mechanism to manage stressful events among parents of children with ASD.

A considerable amount of qualitative literature was available on coping mechanisms among parents of children with ASD. In a qualitative study by Gray (41) on 28 parents of children with ASD (mothers, *n* = 19 and fathers, *n* = 9), the author summarized that the parents used religious faith as their problem-focused coping strategy. In comparison, fewer parents used social withdrawal, family support, and reliance on service providers as their emotion-focused coping strategies.

Lin and colleagues (42) conducted a qualitative case study to investigate the coping mechanism among 17 parents of children with ASD. In contrast, the authors found that seeking support, adjusting to self-change, and developing treatments for their children were problem-focused and emotion-focused coping strategies used by the parents. Furthermore, a qualitative study by Luong et al. (43) reported that parents used nine coping mechanisms, including spiritual coping, acceptance, redirecting energy, empowerment, social withdrawal, denial, rearranging relationships, changing expectations, and changing the focus. Gona et al. (44), in a recent study, used qualitative methodology based on in-depth interviews and focused group discussion. They found that the parents avoided certain foods to control their children's behaviors and sent them to a boarding school as problem-focused coping strategies. They also found that the parents used religious behaviors as their emotion-focused coping strategy (44).

## Discussion

Numerous studies were conducted to compare coping mechanisms among parents of children with ASD and parents of children with other developmental disorders. In this regard, Wang, Michaels, and Day (45) conducted a descriptive study to identify and compare coping mechanisms between parents of children with ASD and parents of children with developmental disorders. A total of 368 parents were assigned to one of four groups: Group 1 (ASD, *n* = 137), Group 2 (mental retardation, *n* = 135), Group 3 (physical disabilities, *n *= 44), and Group 4 (developmental disabilities, *n* = 52). They assessed the coping mechanisms using the COPE questionnaire, a self-report questionnaire containing 60 items and 15 subscales on a four-point Likert scale. The authors found that five of the fifteen strategies were frequently used by the parents: active coping, positive reinterpretation and growth, acceptance, planning, and suppression of competing activities. Also, the results indicated that planning was more frequently used by parents of children with ASD as the coping mechanism than by parents of children with other disabilities. Lai and colleagues (33) compared coping mechanisms between parents of children with ASD and parents of children with typical development. A total of 136 parents were assigned to one of the two groups: Group 1 (ASD, *n* = 73) and Group 2 (typical development, *n* = 63). In their study, the coping mechanism was assessed using the Brief COPE questionnaire, a self-report questionnaire containing 28 items on a four-point Likert scale. The study results indicated that active avoidance was more frequently used by parents of children with ASD as the coping mechanism than by parents of children with typical development. Overall, to date, debate continues about the best coping mechanism to deal with stressful situations among parents of children with ASD. To sum up, there has been little agreement and continuous debate on the best coping mechanism in dealing with the stressful situation among parents of children with ASD. Two types of coping mechanisms, namely problem-focused and emotion-focused coping mechanisms, are worthy of discussion. The study by Zablotsky et al. (34) indicated that emotion-focused coping was most frequently used by parents of children with ASD, which is in agreement with studies by Sivberg (35), Twoy et al. (36), Benson (37), and Smith et al. (38). Also, Pepperell et al. (40) stated that parents of children with ASD used problem-focused coping mechanisms frequently, which is congruent with the report by Kiami and Goodgold (39). Regarding qualitative findings, Gona et al. (44) found that both problem-focused and emotion-focused coping mechanisms were used by parents of children with ASD, which is supported by Lin et al. (42) and Luong et al. (43). In contrast, Gray (41) found that parents used only emotion-focused coping.

*Different coping mechanisms used by mothers and fathers* *of children with ASD*

To date, only a few studies have investigated coping mechanisms used by fathers and mothers of children with ASD. In particular, mothers of children with ASD were reported to employ emotion-focused coping, such as social and emotional support and spiritual strategies (6). In contrast, the reported emotion-focused coping used by fathers was more to suppress frustrations and avoid family problems (33,46). 

Hastings et al. (46) reported that mothers of children with ASD used emotion-focused coping more frequently than fathers. Lee (47) also reported a similar result to Hastings et al.’s results (46). A study was conducted by Willis et al. (48) to explore different coping mechanisms among parents of children with ASD. A total of 89 participants (fathers, *n* = 43 and mothers, *n* = 46) were included in the study, and their coping mechanisms were assessed using the Brief COPE scale, a self-administered instrument containing 14 subscales on a Likert scale ranging between 0–4. The study results showed that the mothers used emotion-focused coping, while the fathers used problem-focused coping. In another recent study, Luque Salas et al. (49) examined the difference in coping mechanisms implemented by mothers and fathers of children with ASD. In their study, 129 parents were included, which were 64 fathers and 65 mothers. They used the Coping Strategy Inventory (SCI), a self-reported measure containing 40 items ranging between 0-5. The study revealed that the fathers used avoidance strategies as their problem-focused coping strategy, whereas the mothers used social support as their emotion-focused coping strategy. 

In summary, so far, there has been little discussion on differences in coping mechanisms used by fathers and mothers. Luque Salas et al. (49) indicated that emotion-focused coping was more frequently used by mothers of children with ASD, consistent with the findings obtained by Lai et al., (33), Hastings et al. (46), Lee, (47), and Willis et al. (48). On the other hand, the same study indicated that fathers of children with ASD used problem-focused coping as their coping mechanism, which agrees with previous studies by Lai et al., (33), Hastings et al. (46), Lee, (47), and Willis et al. (48). Nevertheless, far too little attention is paid to identifying differences in coping mechanisms used by mothers and fathers of children with ASD.

## In Conclusion

This review focused on identifying types of coping mechanisms and different coping mechanisms between mothers and fathers of children with ASD. Previous literature studies provided a consistent picture to agree that parents use two main coping mechanisms when caring for their child with ASD, namely, problem-focused coping and emotion-focused coping. In addition, previous studies showed that emotion-focused coping was widely demonstrated by mothers, whereas fathers used problem-focused as a coping mechanism. As a result, this review offers essential insights into research as a key to developing an intervention support program that mainly focuses on coping mechanisms among fathers and mothers of children with ASD and improving the adaptation to stressful events.

## Author’s Contribution

All the authors supported the preparation and writing of the manuscript and approved the final manuscript as submitted. All the authors agreed to be accountable for all aspects of the work, ensuring that questions related to the accuracy or integrity of any part of the work were appropriately investigated and resolved. 

## Conflicts of interest

The authors declare no conflicts of interest regarding a review publication.
